# Improving the risk management process in quality management systems of higher education

**DOI:** 10.1038/s41598-024-53455-9

**Published:** 2024-02-17

**Authors:** Oleg Bazaluk, Artem Pavlychenko, Olena Yavorska, Olha Nesterova, Vitaliy Tsopa, Serhii Cheberiachko, Oleg Deryugin, Vasyl Lozynskyi

**Affiliations:** 1 Belt and Road Initiative Center for Chinese-European Studies (BRICCES), Guangdong University of Petrochemical Technology, Maoming, 525000 China; 2 Department of Ecology and Technologies of Environmental Protection, Dnipro University of Technology, Dnipro, 49005 Ukraine; 3 Department of Labor Safety and Civil Security, Dnipro University of Technology, Dnipro, 49005 Ukraine; 4 Department of Philosophy and Pedagogics, Dnipro University of Technology, Dnipro, 49005 Ukraine; 5 Department of Management and Economics, International Institute of Management, 10/12B Shuliavska Str., Kyiv, 04116 Ukraine; 6 Department of Transportation Management, Dnipro University of Technology, Dnipro, 49005 Ukraine; 7 Department of Mining Engineering and Education, Dnipro University of Technology, Dnipro, 49005 Ukraine

**Keywords:** Risk factors, Human behaviour

## Abstract

The purpose of this paper is to improve the risk management process in the quality management system of higher education, taking into account the hazardous factors that increase the probability of occurrence and severity of consequences of undesirable events, as well as favorable factors that reduce the probability of occurrence and severity of consequences of hazardous events. The basis of risk management in the quality management systems of higher education institutions is the “Bowtie” method, which involves six main steps of identifying inconsistency, determining the impact of hazardous and favorable factors according to the impact group, ranking hazardous and favorable factors, calculating risk, substantiating precautionary measures and checking calculations. To rank hazardous and favorable factors, the authors used the “Decision Making Trial and Evaluation” method (hereinafter—DE-MATEL), which is based on paired comparison and decision-making tools based on graph theory. An improved process is proposed for risk assessment, which differs from the known ones by the presence of the identification of the cause-and-effect relationship “hazard (inconsistency)-hazardous event-consequences”, identification of hazardous and favorable factors of the internal and external environment that affect the probability and/or the degree of severity of a hazardous event—the appearance of an inconsistency, which is carried out after the inconsistency has been determined; determination of causal hazardous and favorable factors by an acceptable method. Registers of inconsistencies (hazards), hazardous and favorable factors have been developed and proposed based on the requirements for accreditation of educational programs and the international standard ISO 9001:2015, which will allow, based on a risk-oriented approach, to provide a basis for setting the goals of a higher education institution under martial law in order to guarantee effective implementation of the mission and strategy. They are proposed for decision-making in the quality management systems of educational organizations on the substantiation of precautionary or corrective measures based on ranking the risks from identified inconsistencies, which are determined taking into account the impact of the entire set of identified significant hazardous and favorable factors. The value of this paper is to improve the quality risk management process in educational organizations, taking into account the impact of hazardous and favorable factors, and to develop an appropriate step-by-step algorithm of actions and a risk assessment form.

## Introduction

Higher education has entered the era of digitization, in which the central concept is quality^1^, which is a rather complex category, as it determines the standard of living of both an individual and society as a whole. Therefore, each educational institution plays a special role in shaping the future development of the country^2^. This requires the developers of educational policy to constantly raise the requirements for the quality of higher education by organizing the effective work of the accreditation agency, ranking universities and their financial support^3,4^. Thus, the accreditation process serves as a road map for creating and maintaining a culture of high-quality training of students, through the assessment of various external and internal factors, such as leadership, management, resources, teaching methods, evaluation, etc.^5^. In addition, accreditation helps higher education institutions to unify their vision through the mission, goals and strategic direction of development, which is at the basis of effective management of the system of students training. However, the low level of corporate culture, uneven distribution of workload, outdated teaching methods (there are quite significant dynamic changes in the world), insufficient attention to innovation and transformation are constant challenges, both for the heads of higher education institutions and for a teacher, in terms of ensuring quality of educational program, which requires a responsible attitude to the distribution of financial and human re-sources. On the other hand, modern trends show that ensuring the appropriate quality training of students requires the university to agree on the mission, strategic goals, policy, culture and key performance indicators^6–9^. As a result, some higher education institutions in order to improve efficiency are implementing an education quality management (QM) system based on international standards (Table 1), which focuses on meeting the requirements of stakeholders, managing resources, the teaching and learning process, as well as supporting all areas of university life affecting the quality of providing educational services.

**Table 1:** International standards of the quality management system in education.

No.	Standard/Title	Country	Description
1	ISO 9001:2015 Quality management systems—Requirements	International Organization for Standardization	The standard is adapted to the so-called high-level structure, a basic structure with single main texts for the central requirements for management systems, as well as common designations and definitions, which was the basis for all management system standards. The standard considers the “Context of the organization”, that is, the internal and external environment of the company, the requirements of the relevant stakeholders, the emphasis is placed on the responsibility of the top management, a risk-oriented approach is introduced, which replaces, among other things, the so-called preventive measures, hazard identification, risk assessment and opportunities
2	ISO 21001:2018 Educational organizations Management systems for educational organizations Requirements with guidance for use	International Organization for Standardization	Specifies the requirements to management systems for educational organizations, used by educational organizations to develop and implement quality management systems that provide education services, the only tool for managing the organization's processes based on risk management
3	HB 90.7-2000 (R2016) Education and Training Guide to ISO 9001»	Australia	In 2016, the standard was revised and brought into line with ISO 9001:2015. It contains guidelines for the application of ISO 9001:2015 in educational institutions. This standard explains the application of ISO 9001:2015 in terms understandable to education professionals. The standard also provides the most typical examples of requirements implementation
4	ASQ Z1.11-2011 (R2016) Quality management system standards—Requirements for education organizations	USA	In 2016, the standard replaced the second edition of the standard (ANSI/ASQC Z1.11:2002). The standard contains explanations and recommendations for fulfilling the requirements of Q9001 (the American counterpart of the international standard ISO 9001) in educational institutions
5	Esquema 1 IRAM 30000 Guia para la interpretación ISO 9001:2000 en la educacion	Argentina	An Argentine standard that explains the application of the requirements of ISO 9001 to educational institutions and the interpretation of these requirements in terms used in education

The basis of the mentioned standards is a risk-oriented approach requiring higher education institutions to plan and perform certain actions regarding the consideration of risks and opportunities, which is the “foundation” of the effectiveness of the quality management system, the achievement of improved results and the prevention of negative impacts^10,11^. Hence, there is a need to develop a clear, efficient and effective risk management process, which is an urgent task due to the multifaceted nature of the problems faced by universities and a significant number of approaches that can be applied to solve them. It should be noted, based on a number of scientific studies^12,13^, that the implementation of quality management system in a higher education institution based on 9001:2015 requirements leads to several unresolved issues: for example, there is no clear methodology of risk management specifically in educational organizations with the specificity of training applicants whose quality of skills cannot be simultaneously tested (for example, new knowledge are found, a different vision that does not correspond to existing scientific paradigms^14^), or there are no recommendations regarding the level of organizational culture at which the requirements of the specified standard will be effective and will be implemented.

Analysis of scientific research over the past years has shown an increase in interest in ensuring the sustainability of educational institutions in emergency situations, which is connected with the global spread of the pandemic and the need to transfer education to a distance format^15^. The largest number of studies is devoted to the identification of risks during the provision and organization of distance learning. In particular, the authors^16^ paid attention to the stability of communication, the adverse effect of technological zones near computers, the lack of physical contact between teachers and students, and the overloading of students with online tasks.

At the same time, in the analysed work, the conclusions drawn were based on an online questionnaire of the participants of the educational process, which contained a limited number of hazardous factors (hereinafter—HF), which does not allow us to talk about a comprehensive assessment of the problem. In other studies, attention was focused on the study of negative consequences when using information technologies in the management system of an educational institution^17^. The authors, using their own eight-step algorithm, analysed problem areas of information systems and developed recommendations for reducing the negative consequences of various failures. However, the paper considered only the area of management of the educational institution, while the educational process itself and its provision was omitted.

We note the fact that in the domestic space, a fairly significant number of publications are devoted to the risks and challenges of reforming both secondary and higher education, taking into account the need for digitalization of the educational process^17–19^. For example, in the papers^20,21^, the authors analysed the risk factors and identified the biggest of them: low-quality Internet and lack of information and communication tools for learning, which is the biggest threat to distance learning. Another paper^22^ systematized and singled out the groups of external and internal risks of educational activity, comprehensively revealed the system of external risks and proposed possible mechanisms for responding to them, outlined the range of losses (economic, social, political and pedagogical) that are the result of the occurrence of risky events. And also the main conclusion drawn from the publication is the need for the effective implementation of educational activities in the field of higher education, for higher education institutions to timely identify and identify risks, which will help reduce the level of uncertainty in the activities of educational institutions, to determine the direction of movement on which it is necessary to focus managerial, personnel, methodological, research, financial, technological and organizational resources. The authors of the article^23^ revealed the need to apply a risk-oriented approach to the implementation of the quality management system of higher education institutions, and also systematized groups of internal risks in the course of operational processes in the activities of higher education institutions and proposed possible mechanisms for responding to them.

The conducted analysis shows that it is possible to apply a risk-oriented approach to the management processes of educational institutions only from the position of making managerial decisions to achieve quality goals, when the manager determines opportunities and associated risks^24^. However, there are issues related to the limitation of resources, which requires the elimination of existing deviations or errors in risk assessments that will lead to unwanted financial losses. Moreover, in the processes themselves, risk is proposed to be determined in relation to potential events that are influenced by groups of different factors or their combination^25^, and therefore, in order for organizations to carry out a qualitative or quantitative assessment of risk and make appropriate management decisions, it is necessary to develop an appropriate management process risks. The authors of the publication^26^ suggest using the improved AHP methodology to assess the risks and opportunities of the quality development of higher education from the point of view of ISO45001:2018 as the case study of higher education institutions in China.

Thus, the conducted research analysis shows that there are several directions of quality assurance in higher education institutions: curricula, criteria and rules for assessing students, quality of teachers and educational process, quality of material-technical and information resources, as well as collection, analysis and use of information^27^. At the same time, ensuring the quality provision of educational services involves systematically identifying and managing the various processes encountered in educational organizations^28^. This is mainly done by applying a management system based on the PDCA cycle with a general focus on risk-oriented thinking, the key element of which is the implementation of necessary actions to achieve the planned measures and results based on risk management, requiring the effective process development.

The aim of this paper is to improve the risk management process in the quality management system of higher education in accordance with the requirements of 9001:2015, ISO 21001:2018 and other standards, taking into account HFs, which increase the probability of occurrence and severity of consequences, as well as FFs, which reduce the probability of occurrence and severity of consequences from hazardous events.

## Materials and methods

The most common approach to risk management in the quality systems of any organization, which is the basis for developing an action plan to achieve the desired result and identifying the resources required for the effective and efficient functioning of each process, is the “Bowtie” method^29^.

This is the most common model, the popularity of which is attributed to the convenience and simplicity of presenting the cause-and-effect relationship between hazard, hazardous event and consequences. The visualisation of this model helps to clearly demonstrate the risk management process by determining the number of barriers (protective or precautionary measures) that are placed on the path between a hazard and a hazardous event, as well as between a hazardous event and its consequences. The number of barriers makes it possible, on the one hand, to estimate protective or precautionary measures for occupational safety, and on the other hand, to influence the probability of the hazardous event occurring. The latter is determined by the “as low as reasonably practicable” (ALARP) principle, according to which the residual risk level should be reduced as much as possible^30^.

**Figure 1:**
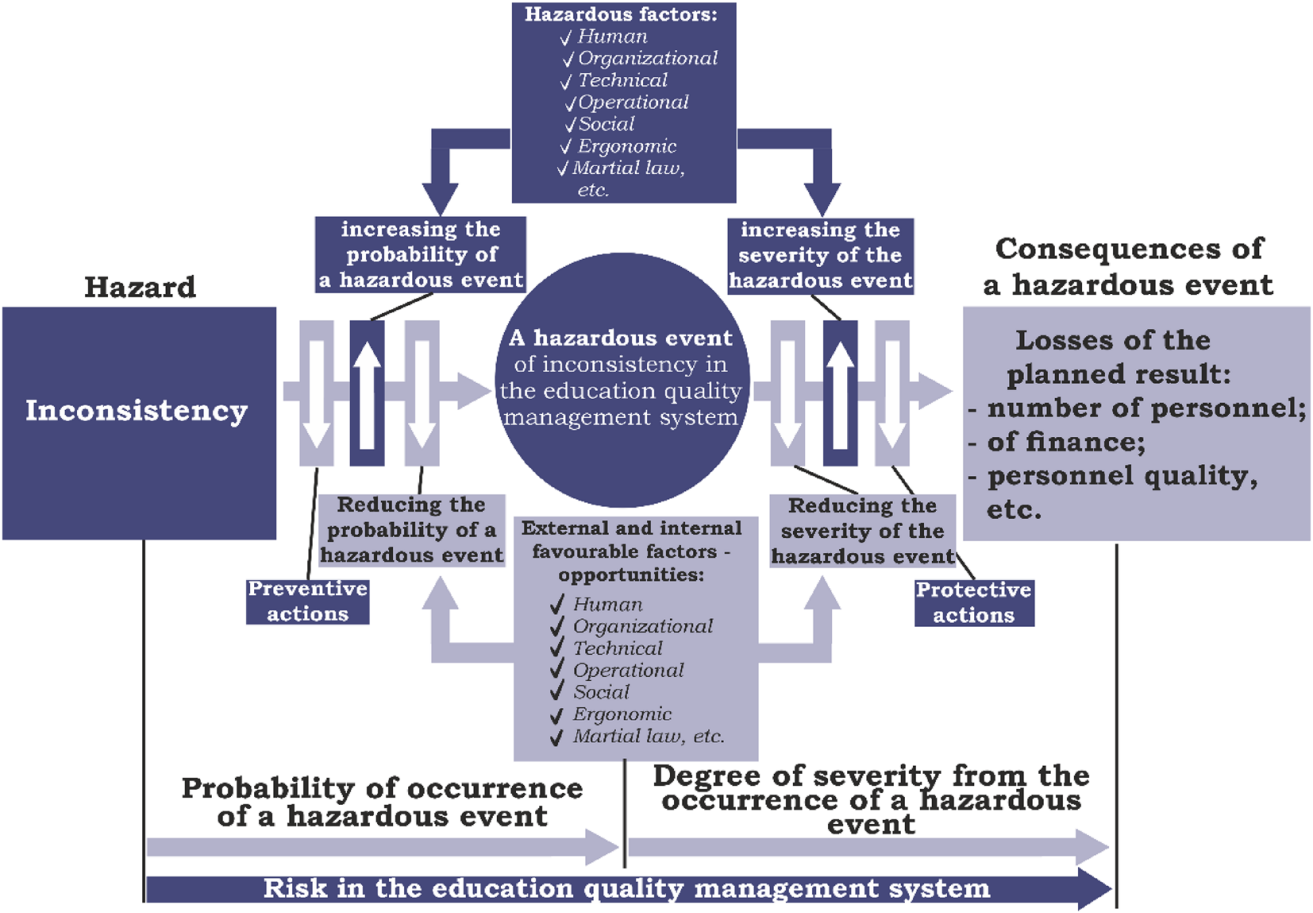
Risk management procedure in educational quality management systems based on the “Bowtie” model.

It makes it possible, in general, to understand the reasons for the occurrence of a hazardous event from the presence of certain inconsistencies in any process or threats (challenges) to the organization and to assess the consequences of their occurrence, which is the basis of risk calculation. This method also allows you to justify the financial costs of the risk management process based on the analysis of the efficiency of the created protective barriers that are placed on the path from inconsistency to a hazardous event, as well as between a hazardous event and its consequences (Fig. 1).

**Figure 2:**
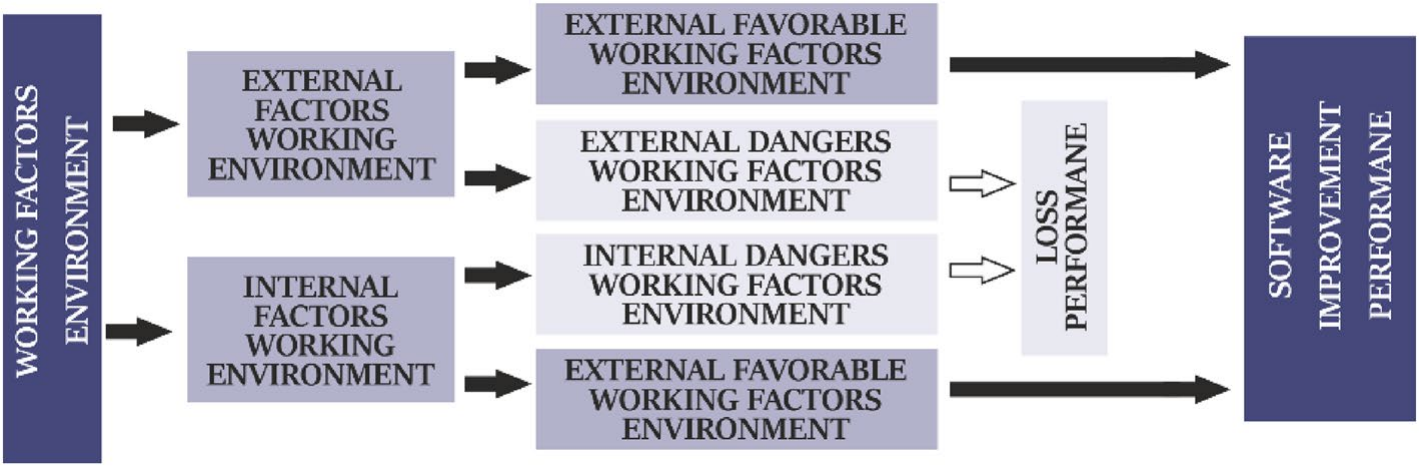
Hazardous and favorable factors that lead to an increase in the probability of a hazardous event occurring.

**Figure 3:**
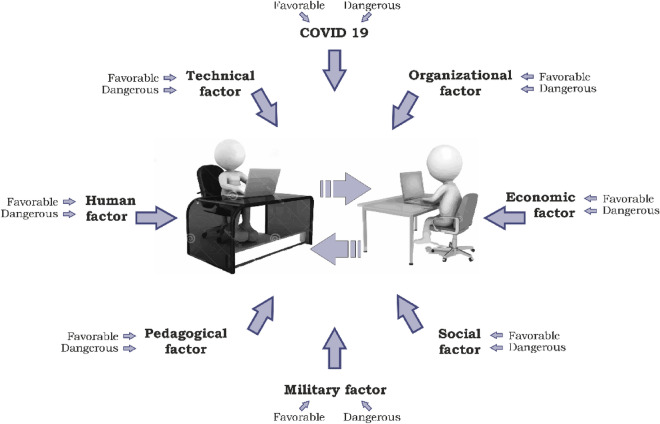
Hazardous and favorable factors that lead to an increase in the probability of a hazardous event occurring.

This approach is characterized by the ability to take into account the influence on a change (increase or decrease) in the probability of occurrence and the severity of the consequences of a hazardous event of various external and internal hazardous and favorable factors (Fig. 2), which are proposed to be divided into seven groups: human, technical, organizational, economic, social and pedagogical, martial law (Fig. 3). In this case, hazardous factors (HF) influence an increase in the probability of occurrence and/or severity of consequences of a hazardous event and, as a result, an increase in risk, which is a loss of effectiveness, especially in quality management systems. Favorable factors (FF) have an impact on reducing the probability of occurrence and/or severity of consequences of a hazardous event and, ultimately, on reducing the risk together with protective or precautionary actions (measures), which is the improvement of effectiveness, especially in the quality of providing the educational process.

**Figure 4:**
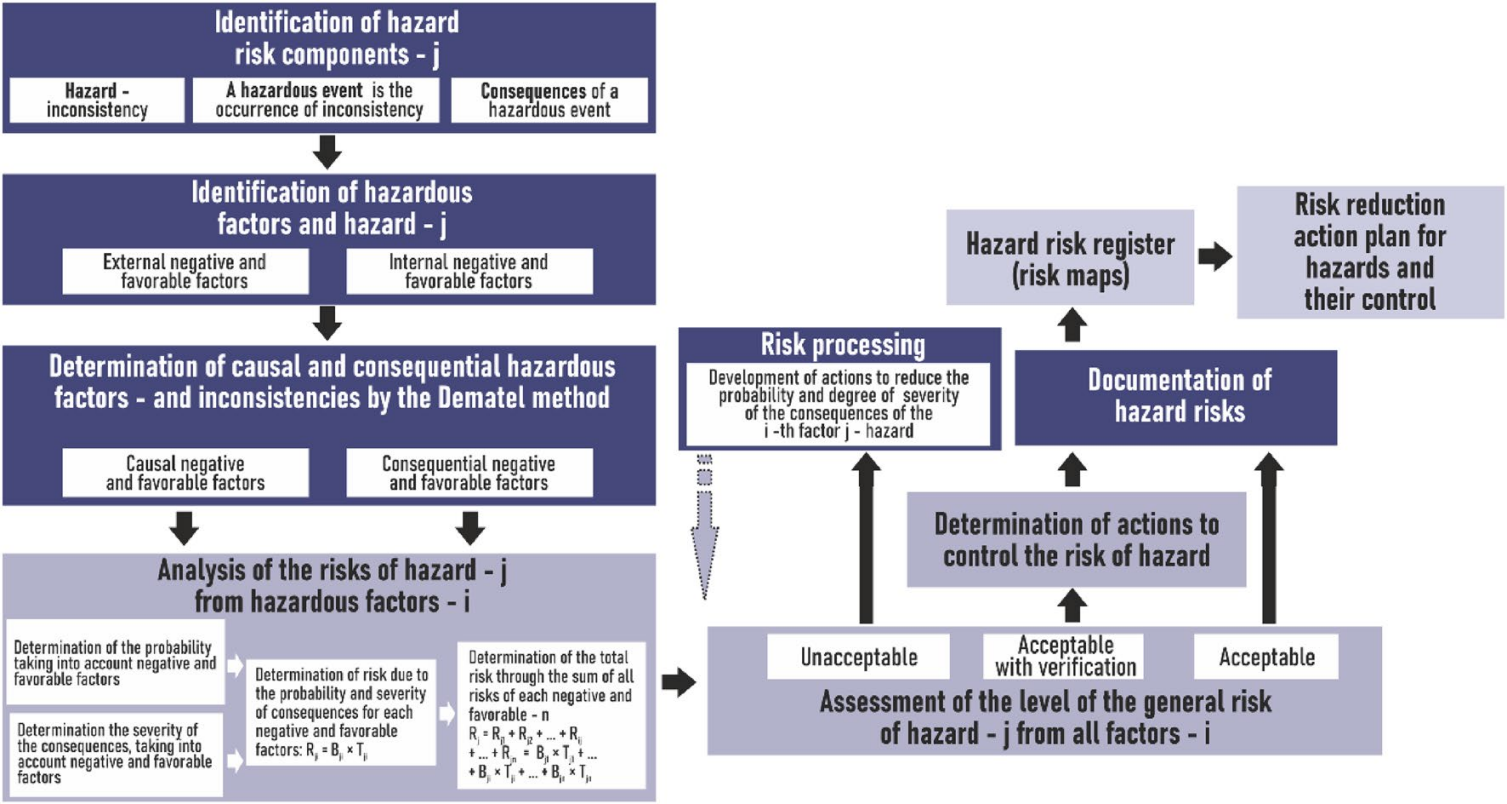
Risk management process.

An improved risk management process consisting of six main steps is proposed for risk assessment (Fig. 4). The main difference between the proposed process and the known ones^27,31–33^ is identification of the cause-and-effect relationship “hazard (inconsistency)-hazardous event-consequences”, identification of hazardous and favorable factors of the internal and external environment, which affect the probability and/or degree the severity of the hazardous event—the appearance of an inconsistency, which is carried out after the inconsistency has been determined; determination of causal (ignoring consequential) HFs and FFs by an acceptable method (for example, DEMATEL or ANP).

The first step is devoted to determining the cause-and-effect relationship between “hazard (inconsistency)-hazardous event-consequences” in the quality system of higher education, which can lead to the occurrence of a hazardous event under certain conditions, for example, a low level of training of higher education students. The result of inconsistencies can be seen as the loss of the image of the university, the number of applicants, financial support, personnel potential, and others (Table 2). This step requires the creation of an appropriate catalogue of hazards (inconsistencies), which are violations of the requirements of legislation in the field of education (higher education standards). The basis of its creation is a survey of interested parties, who are invited to provide an answer that affects the process of quality training of students. For example, the Table 3 provides the list of inconsistencies (threats) compiled on the basis of the requirements for accreditation of educational programs.

**Table 2:** Examples of identification of the cause-and-effect relationship “hazard-hazardous event-consequences”.

No.	The hazard which is inconsistency	Hazardous event (which is associated with the inconsistency)	Consequences (losses)
1	Non-fulfillment of requirements for accreditation of educational programs	Loss of accreditation of educational programs	Decrease in income, loss of reputation
2	Failure to comply with requirements to evacuate students to a bomb shelter during an air raid alert	Missiles and drones of the aggressor, or parts of them shot down by the air defence system of the Armed Forces of Ukraine, hit the building of an educational institution	Loss of health and life of teachers; students Loss of reputation of the educational institution
3	Loss of reputation of the educational institution	Employers’ refusal to employ graduates of an educational institution	Unemployed graduates of an educational institution Loss of reputation of the educational institution

**Table 3:** Fragment of the list of hazards (inconsistencies) in educational programs.

No.	Educational program criteria	Designation	Hazard-inconsistency
1	Design and goals of the educational program	H_1_	The goals of the educational program are not determined taking into account the needs of the interested parties
		H_2_	The educational program does not provide an opportunity to achieve learning outcomes defined by the standard of higher education
2	Structure and content of the educational program	H_3_	In the educational program, the structural and logical scheme does not reflect the relationships of the educational components
		H_4_	The content of the educational program does not correspond to the subject area of the specialty determined for it
		H_5_	The structure of the educational program does not provide for the possibility of forming an individual educational trajectory
		H_6_	The educational program and curriculum do not provide for practical training of applicants
3	Access to educational program	H_7_	The rules of admission to study under an educational program do not take into account its peculiarities
4	Learning and teaching according to the educational program	H_8_	Forms and methods of training do not contribute to the achievement of the goals and program results of training stated in the educational program
			A combination of teaching and research is not provided during the implementation of the educational program
			Training and teaching within the educational program is not carried out in compliance with the requirements of the legislation
5	Control, evaluation of students and academic integrity	H_9_	Forms of control and evaluation criteria for higher education students are unclear and not understandable
		H_10_	The educational institution does not have clear and understandable policies, standards and procedures for compliance with academic integrity
		H_11_	The forms of attestation of higher education students do not meet the requirements of the higher education standard
6	Human resources	H_12_	Procedures for the competitive selection of teachers are non-transparent
		H_13_	The educational institution does not involve professionals-practitioners to teach for the educational program (in particular, conducting in-class activities)
		H_14_	Teachers do not have appropriate qualifications and/or professional experience and are unable to ensure the implementation of educational program
7	Educational environment and material resources	H_15_	Financial and material and technical resources do not guarantee the achievement of the educational program goals and program objectives
		H_16_	The educational environment does not provide an opportunity to satisfy the needs and interests of students
		H_17_	The educational institution does not provide access to the appropriate infrastructure for teachers and higher education students

In the second step, we determine the HFs and FFs, from which we also form the corresponding register (Tables 4, 5). In the given example, HF hazards (inconsistencies) include: non-fulfilment of the requirements for accreditation of educational programs, non-fulfilment of the requirements for the evacuation of applicants to a bomb shelter during an air raid alert, non-fulfilment of the requirements for traineeships of students at production enterprises—low competence that affect the probability of occurrence and degree of severity losses from this hazard The list of HFs and FFs is determined based on the development of various studies on the problems of management of higher education institutions, surveys of experts in this field, interviews with the institution management and staff. Please note that the list of HFs and FFs in each institution of higher education may differ, based on the specific circumstances that have developed in it. In addition, such a determination of HFs and FFs must be made for each hazard (inconsistency).

**Table 4:** Example of HF risk in the quality system of an educational organization.

No.	GF	HF risk	
1	Human	A_1_	Inadequate psychological state of teachers and students
		A_2_	Lack of motivation for the educational process
		A_3_	Unpreparedness of teachers to work in an innovative educational environment
		A_4_	The complexity of online communication between the teacher and the applicant
		A_5_	Low digital literacy of teachers and students
		A_6_	Decrease in social morality, spirituality, culture of behaviour of teachers
2	Technical	A_7_	Low-quality software of the educational process
		A_8_	Low level of technical support of the educational process
		A_9_	Lack of high-quality and stable Internet connection
		A_10_	Lack of high-quality educational content on distance education platforms
		A_11_	Low-quality provision of computer and multimedia equipment
		A_12_	Non-compliance with sanitary standards at workplaces
		A_13_	Lack of updating, repair and maintenance of technical teaching aids
3	Organiza-tional	A_14_	Imperfection of mechanisms for the implementation of the educational process
		A_15_	The complexity of online communication between the teacher and the student
		A_16_	Lack of personnel, demotivation of personnel, decrease in competence
		A_17_	Insufficient monitoring of teachers' psychological-pedagogical competence development
		A_18_	Inefficient management of scientific, research, educational activities
		A_19_	Inefficiency of the system and mechanisms for academic integrity support
		A_20_	Inefficient personnel policy, violation of corporate culture
4	Economic	A_21_	Lack of working capital to support the educational process
		A_22_	Weakness of marketing policy in conditions of competition
		A_23_	Insufficient state funding of educational activities
		A_24_	Lack of activity of students and teachers in grant support
		A_25_	Ineffective monitoring of the financial potential of the educational institution
		A_26_	Inadequate system for effective cost control
5	Social	A_27_	Ineffective institutional support for employee welfare
		A_28_	Inadequate salary of teachers and staff
		A_29_	Decrease in student participation in university events
		A_30_	Inefficient mechanisms for settling conflict situations
6	Pedagogical	A_31_	Absence (inefficiency) of a holistic system of education, physical, moral and spiritual development of students in educational programs
		A_32_	Inadequate structure and content of the educational program to the needs of the labour market
		A_33_	Lack of innovative and progressive teaching methods
		A_34_	The imperfection of the system for monitoring and evaluating the knowledge of the students
		A_35_	Lack of feedback from the teacher to the students
		A_36_	The imperfection of the mechanisms of involvement of stakeholders in the educational process
		A_37_	Inefficient mechanisms for improving educational programs
		A_38_	The imperfection of the educational process planning system
7	Military	A_39_	Air alarms that interrupt classes
		A_40_	Missile threats that cause neurotic states
		A_41_	Power outages, impossibility of classes
		A_42_	Placement of classrooms near strategic objects
		A_43_	Lack of internet connection, impossibility of online communication
		A_44_	A state of frustration due to a constant flow of threatening information
8	COVID-19	A_45_	Absence of the possibility of conducting laboratory and practical classes offline
		A_46_	Lack of live communication between the teacher and the student during classes
		A_47_	A state of frustration due to the constant worry of getting sick
		A_48_	Social isolation

*GF* group of factors.

**Table 5:** Example of FF risk in the quality system of an educational organization.

No.	GF	FF risk	
1.	Human	B_1_	Adequate psychological state of teachers and applicants
		B_2_	Motivation for the educational process
		B_3_	Readiness of teachers to work in an innovative educational environment
		B_4_	Availability of online communication between the teacher and the applicant
		B_5_	High digital literacy of teachers and applicants
		B_6_	Increase in social morality, spirituality, culture of behaviour of teachers
2.	Technical	B_7_	High-quality software of the educational process
		B_8_	High level of technical support of the educational process
		B_9_	Availability of high-quality and stable Internet connection
		B_10_	Availability of high-quality educational content on distance education platforms
		B_11_	High-quality provision of computer and multimedia equipment
		B_12_	Compliance with sanitary standards at workplaces
		B_13_	Updating, repair and maintenance of technical teaching aids
3.	Organizational	B_14_	Perfection of mechanisms for the implementation of the educational process
		B_15_	Easy online communication between the teacher and the applicant
		B_16_	Availability of personnel, motivation of personnel, increase in competence
		B_17_	Availability of monitoring for teachers' psychological-pedagogical competence development
		B_18_	Efficient management of scientific, research, educational activities
		B_19_	Efficient system and mechanisms for academic integrity support
		B_20_	Efficient personnel policy, violation of corporate culture
4.	Economic	B_21_	Availability of working capital to support the educational process
		B_22_	Availability of marketing policy in conditions of competition
		B_23_	Sufficient state funding of educational activities
		B_24_	Availability of activity of applicants and teachers in grant support
		B_25_	Effective monitoring for the financial potential of the educational institution
		B_26_	Availability of adequate system for effective cost control
5.	Social	B_27_	Effective institutional support for employee welfare
		B_28_	Adequate salary of teachers and staff
		B_29_	Increase in student participation in university events
		B_30_	Efficient mechanisms for settling conflict situations
6.	Pedagogical	B_31_	Availability of efficient holistic system of education, physical, moral and spiritual development of applicants in educational programs
		B_32_	Adequate structure and content of the educational program to the needs of the labour market
		B_33_	Availability of innovative and progressive teaching methods
		B_34_	Perfection of the system for monitoring and evaluating the knowledge of the applicants
		B_35_	Availability of feedback from the teacher to the applicants
		B_36_	Perfection of the mechanisms of involvement of stakeholders in the educational process
		B_37_	Efficient mechanisms for improving educational programs
		B_38_	Perfection of the educational process planning system

*GF* group of factors.

**Figure 5:**
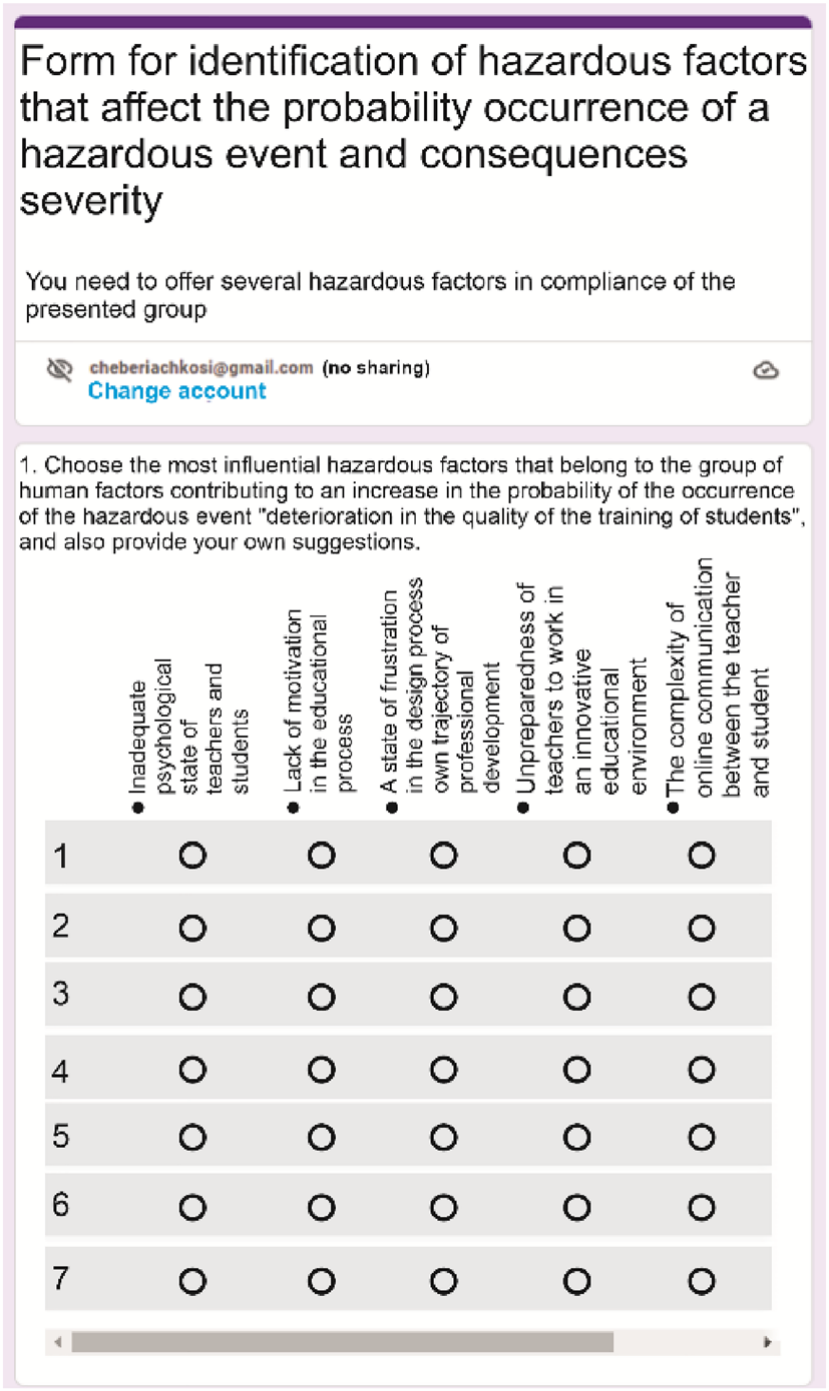
Fragment of a form for determining the influence of hazardous and favorable factors that increase the probability of a hazardous event occurring from a specific hazard (inconsistency).

In order to identify the impact of HFs and FFs on the probability and severity of consequences of a hazardous event occurring due to one or another inconsistency, a process of surveying of all interested parties (teachers, students, employers, management of a higher education institution, technical workers, etc.) is carried out. They are offered to choose one or another HF and FF in an online questionnaire (Fig. 5).

The above excerpts are from a survey conducted at Dnipro University of Technology, located in the city of Dnipro, Ukraine, in the period from February to June 2022. The survey involved 112 people (43 men and 69 women) aged between 18 and 43. The majority of participants (70%) are university lecturers, 22% are applicants and 8 are employees of various departments. In this case, the average work experience of teachers is 12.4 years. Their scientific field of activity is related to mining (33.2%), construction (26.8%), service sector (31.4%), economics (4.2%), ecology (14.6%).

The result of such a procedure is the formation of a register (Table 6) of the most influential HFs and FFs on the determined inconsistency, which are then ranked to identify the most important ones. This procedure will be conducted by several recognized experts representing the management of the institution, teachers and at least 5 applicants.

**Table 6:** Forms for the register of HFs and FFs that increase/decrease the probability of a hazardous event occurring.

Identification of the causal relationship “Hazard-hazardous event-consequences”			HF	FF
Inconsistency	Hazardous event	Negative consequences		
Non-compliance (e.g. failure to meet any requirement or accreditation requirements)	Loss of accreditation	Loss of income from training according to this accreditation	A_1_	B_1_
			…	
			A_i_	B_i_
			…	
			A_n_	B_n_

In the third step, we study the interaction of HFs and FFs with the identified hazards (inconsistencies) and use the “Decision Making Trial and Evaluation” (DEMATEL) method, which is based on paired comparison and decision-making tools of graph theory^34,35^, which will allow us to transform cause-and-effect relationship in structural-visual models and identify and understand the interdependencies between different HFs and FFs that will lead to negative consequences. Relationships between influencing factors are made on the basis of paired comparison.

This case study involved five experts certified as internal or external auditors of quality management systems in accordance with the requirements of the ISO 9001:2015 international standard and the ISO 19011:2018 provisions; work experience in an educational organization is for at least 5 years; degree of education—the level is not lower than Doctor of Philosophy; they also have relevant documents certifying their training and knowledge of standards: standards and recommendations of the European Higher Education Area ESG-2015; ISO 21001:2018 ”Educational organizations—Management systems for educational organizations—Requirements with guidance for use”; 31000:2018 “Risk management. Principles and guidance”, and knowledge of the expert activity specifics; criteria and indicators of the effectiveness of an expert on the quality of education in EU countries . They are experienced in conducting expert examinations and audits.

To determine the degree of impact between various negative (positive) factors, criteria with a five-level scale are used, consisting of verbal expressions and corresponding fuzzy numbers (Table 7).

**Table 7:** Verbal expressions and corresponding fuzzy numbers^36–38^.

Final equivalent	Description	Fuzzy equivalent		
Very high impact	VH	0.75	1	1
High impact	H	0.5	0.75	1
Low impact	L	0.25	0.5	0.75
Very low impact	VL	0	0.25	0.5
Zero impact	NO	0	0	0.25

The *n* × *n* pair comparison matrix is formed according to experts using the paired comparison method, which is a natural way and is consistent with human intuition and general way of thinking^39^. In this case, a data consistency check is applied using the following formula:

$$ \left( \% \right) = \frac{1}{{m\left( {m - 1} \right)}}\sum\limits_{i = 1}^{m} {\sum\limits_{j = 1}^{m} {\left( {\frac{{\left| {d_{ij}^{k} - d_{ij}^{k - 1} } \right|}}{{d_{ij}^{S} }}} \right) \times 100\% } } , $$

where *m* is criteria number, *k* is number of experts, *d* is the average value of the direct influence of criterion *i* on j. If the average consensus gap coefficient value is less than 0.05, we consider the expert’s assessment as stable^40^.

Variable *H* stands for number of experts;* n* is the number of considered criteria. The comparison between two factors *i* and *j* by expert *k* is shown as $b_{ij}^{k}$. According to the fuzzy theory in Table 7, the value of “zero impact” is 0, “very low impact” is 1, “low impact” is 2, “high impact” is 3, and “very high impact” is 4. As a result, with an independent assessment of each expert, the response matrix is formed by Eq. (1). Given that each component does not affect itself, the diameter components of each response matrix $B_{{}}^{(k)}$ are equal to zero:

1 $$ \left( {1 \le k \le H} \right)B_{{}}^{(k)} = \left[ {b_{ij}^{k} } \right]_{n \times n} . $$

The average matrix $A = \left[ {a_{ij}^{{}} } \right]_{n \times n}$ has a direct impact on the matrix and the average value of expert ratings, which can be calculated according to Eq. (2). This matrix represents the direct impact of each criterion on the other criteria:

2 $$ a_{ij} = \frac{1}{H}\sum\limits_{K = 1}^{H} {b_{ij}^{(k)} } . $$

Matrix *D* can be calculated using Eqs. (3) and (4):

3 $$ D = \left[ {d_{ij} } \right]_{n \times n} = \frac{A}{S}, $$

4 $$ S = \left( {max_{1 < < 1 < < n} \sum\limits_{j = 1}^{n} {a_{ij} } ,\;max_{1 < < j < < n} \sum\limits_{j = 1}^{n} {a_{ij} } } \right). $$

The matrix *T*_*c*_ can be calculated according to Eq. (5).

In this equation, *I* is the identity matrix. Each element of the matrix *T*_*c*_ can be formed with respect to a fuzzy number $\overline{\tau }_{ij} = \left( {l_{ij}^{t} ,m_{ij}^{t} ,u_{ij}^{t} } \right)$. Given Eqs. (5), (6), (7) and (8).

*D*_*1*_, *D*_*m*_, *D*_*u*_ are all matrices *n* × *n*.

5 $$ T_{C} = D\left( {I - D} \right)^{ - 1} , $$

6 $$ \left[ {l_{ij}^{t} } \right] = D_{1} \left( {I - D} \right)^{ - 1} , $$

7 $$ \left[ {m_{ij}^{t} } \right] = D_{m} \left( {I - D_{m} } \right)^{ - 1} , $$

8 $$ \left[ {u_{ij}^{t} } \right] = D_{u} \left( {I - D_{u} } \right)^{ - 1} . $$

To determine the effect and relevance of the criteria, the matrix *T*_*c*_ must be fuzzy first. Equation (9) will be used so that there are no fuzzy *T*_*c*_ matrices.

The action and relevance of criteria are determined using r and c values. The values of Eqs. (10) and (11) can be calculated:

9 $$ B = \frac{{l_{1} + m_{3} + 2u_{2} }}{4}, $$

10 $$ r = \left[ {r_{i} } \right]_{n \times 1} = \left[ {\sum\limits_{j = 1}^{n} {t_{ij} } } \right]_{n \times 1} , $$

11 $$ c = \left[ {c_{j} } \right]_{n \times 1} = \left[ {\sum\limits_{i = 1}^{n} {t_{ij} } } \right]_{n \times 1} . $$

*r* is the sum of row *i*, and *c* is the sum of column *j* of the matrix *T*_*c*_. *r* shows the total effect of direct and indirect influence of *i* on other criteria, and *c* shows direct and indirect total influence of factor j on other factors.

As a result, *r* + *c* indicates the importance of criterion i in the system. *r* − *c* shows the effect of criterion* i* in the system. If *r* − *c* is positive, the action of criterion* i* belongs to the group of causes, and if *r* − *c* is negative, the effect of the criterion, which belongs to the group of “dependents”.

The next step of the procedure is a risk analysis, which involves determining the probability and degree of severity of a hazardous event from the action of the causative HF. For this purpose, appropriate scales have been determined based on the recommendations of the ISO 73:2018 standard, which are summarised in Tables 8 and 9.

**Table 8:** Probability levels (B) of the occurrence of a hazardous event.

Gradation of the level of influence	The probability level of HE	Characteristics of probability (description)
1	Improbable	The probability is close to zero
2	Practically improbable	It is extremely unlikely that the event will occur within the term
3	Unlikely	It is unlikely, but it may happen once during the term
4	Probable	Happens several times during the term
5	Highly probable	HE occurs frequently during the considered period

**Table 9:** Levels of severity of consequences of (T) HE.

Level of impact	The level of consequences	Characteristics of consequences
1	Insignificant	The training of students takes place in accordance with the regulatory requirements of educational standards, the students have a very high level of training, shortcomings or inconsistencies identified do not affect the quality of training
2	Low	The training of students takes place in accordance with the regulatory requirements of educational standards, the students have a high level of training, the shortcomings or inconsistencies identified have a certain impact on the quality of the students’ training, but their influence is due to the personal qualities of the students
3	Moderate	The training of students takes place in accordance with the normative requirements of educational standards, the students have an average level of training, the shortcomings or inconsistencies identified affect the quality of the students’ training, leading to clear gaps that require further non-essential reorganization of the educational process for the training of students
4	Significant	The training takes place in violation of the requirements of educational standards, the students have an average level of training, shortcomings or inconsistencies identified affect the quality of their training, leading to gaps that require the introduction of appropriate changes in the educational process
5	High	The training of students takes place with significant violations of the requirements of educational standards, the students have a low level of training, shortcomings or inconsistencies identified significantly affect the quality of their training, leading to significant gaps that require retraining

Next, the risk level is calculated for each HF and FF as the product of the probability of a hazardous event (*B*) and the severity of the consequences (*T*) from all predetermined most significant HFs and FFs:

The risk from hazardous factors is determined by the formula:

12 $$ P_{i}^{ + } = B_{i}^{ + } \cdot T_{i}^{ + } . $$

The risk from favorable factors is determined by the formula:

13 $$ P_{i}^{ - } = B_{i}^{ - } \cdot T_{i}^{ - } . $$

The total risk from exposure to HF and FF is determined from:

14 $$ P_{i}^{{}} = P_{i}^{ - } \cdot P_{i}^{ + } = B_{i}^{ - } \cdot T_{i}^{ - } - B_{i}^{ + } \cdot T_{i}^{ + } , $$

where *P*^+^_*i*_*; P*^−^_*i*_ are the risk from the *i-th* hazardous/favorable factor, respectively *B*^+^_*i*_*; B*^−^_*i*_ are the probability of occurrence/non-occurrence of a hazardous event from the *i-th* hazardous/favorable factor; *T*^+^_*i*_*, T*^−^_*i*_ are the severity of consequences of the occurrence/non-occurrence of a hazardous event from the *i-th* hazardous/favorable factor; *P*_*i*_* is* the total risk from exposure to hazardous/favorable factor.

The risk assessment form in the quality system is given in Table 10.

**Table 10:** An example of a form for determining the risk levels from HFs and FFs.

Identification of the causal relationship “hazard-hazardous event-consequences”			Identification of HFs and FFs		Primary analysis is determination of the level of risk for each HF	
Inconsistency	Hazardous event	Negative consequences	HF/FF	Influence on the probability of occurrence of a hazardous event from HF	The severity of the occurrence of the hazardous event of the i-th HF	The level of risk from HF-i
Non-compliance	Loss of accreditation		HF_1_	B_pj1_	T_pj1_	R_pj1_
			HF_2_	B_pj2_	T_pj2_	R_pj2_
			…	…	…	…
			HF_*i*_	B_pji_	T_pji_	R_pji_
			…	…	…	…
			HF_n_	B_pjn_	T_pjn_	R_pjn_
			FF_1_	B^+^п_j1_	T^+^п_j1_	R^+^п_j1_
			FF_2_	B^+^п_j2_	T^+^п_j2_	R^+^п_j2_
			…	…	…	…
			FF_*i*_	B^+^п_ji_	T^+^п_ji_	R^+^п_ji_
			…	…	…	…
			FF_*m*_	B^+^п_jn_	T^+^п_jn_	R^+^п_jn_
			Total OR of hazard *j* from all *n* of HF and FF			Rп_j_ = ∑ R^-^п_j1_ – ∑ R^+^п_j1_

After determining the level of risk (Table 11), solutions are proposed for preventive actions to reduce it. According to the results of determining the risk level, it can be assigned to one of the risk groups:

- I, it is necessary to stop the activity of determining and implementing preventive and protective measures to reduce the risk to an acceptable level;
- II, it is necessary to define and implement preventive and protective measures to reduce the risk to an acceptable level in case of partial cessation of activity;
- III, no risk reduction measures are required, but hazard control is required;
- IV, no risk reduction measures are required and no hazard controls are required.

**Table 11:** Criteria for risk acceptance.

No.	OR assessment	Scores	Risk level
1	Acceptable risk	From 0 to 27	IV
2	Acceptable risk with control	From 27 to 54	III
3	Not acceptable risk	From 54 to 108	II
4	Absolutely unacceptable risk	108–150	I

Very often, the protective measures taken reduce the probability of the risk, but do not eliminate the hazard. In these cases, the probability of risk decreases, but its severity remains unchanged. It is also necessary to consider preventive actions aimed at reducing the severity of the consequences. If the level of risk is totally unacceptable and unacceptable, we understand that it is forbidden to carry out work without changing the conditions and without developing and implementing measures to reduce risks. First of all, preventive and protective measures should be implemented to prevent the realization of the hazard in the HE and/or to reduce the consequences of the HE. Control over the prohibition of works is established. It should be noted that the risk acceptance criteria given in Table 11 are notional and need to be assessed in each organization that tries to implement the described approach, as “they are based on the internal and external context and objectives of the organization, and they may arise from laws, policies, standards and other demands^41^. The most common and flexible framework used for risk criteria divides risks into the three groups mentioned above: “unacceptable area”, “ALARP area” and “acceptable area”^42^.

## Results

Take, for example, an educational training program for second-level Master students, where only HF is considered as an example. But the same approach is applied to FF. The list of its inconsistencies is formed based on the analysis of the results of the internal audit, in accordance with the requirements of ISO 9001:2015 or external accreditation of the educational program by experts of the National Agency for Quality Assurance of Higher Education. The result of the work is a catalogue of inconsistencies (Table 12).

**Table 12:** Fragment of analysis of inconsistencies as a result of examination of the educational program by experts of the National Agency for Quality Assurance of Higher Education.

No.	Criterion	Inconsistencies found by the expert group
1	Criterion 2. Structure and content of the educational program	H_3_. In the educational program, the structural and logical scheme does not reflect the relationships of the educational components
2	Criterion 5. Control measures, evaluation of higher education students and academic integrity	H_9_. Forms of control and evaluation criteria for higher education students are unclear and not understandable
3	Criterion 6. Human resources	H_14_. Teachers do not have appropriate qualifications and/or professional experience and are unable to ensure the implementation of educational program

The next step is to introduce a procedure for surveying all interested parties to identify the most influential HFs that increase the probability of occurrence and the severity of the consequences of a hazardous event. For example, for the specified educational program, taking into account its implementation in the conditions of martial law, the experts chose from the list given in Table 3 twelve HFs, which are shown in Table 13.

**Table 13:** Fragment of the results of the processing of the questionnaire of interested parties to detect the HF, which increase the probability of the occurrence of a hazardous event during martial law.

Identification of the causal relationship “hazard-hazardous event-consequences”			HF	
Inconsistency	Hazardous event	Negative consequences		
Teachers do not have appropriate qualifications	Loss of accreditation		A_1_	Inadequate psychological state of teachers and students
			A_5_	Low digital literacy of teachers and students
			A_10_	Lack of high-quality educational content on distance education platforms
			A_14_	Imperfection of mechanisms for the implementation of the educational process
			A_27_	Ineffective institutional support for employee welfare
			A_35_	Lack of feedback from the teacher to the students
			A_39_	Air alarms that interrupt classes
			A_40_	Missile threats that cause neurotic states
			A_41_	Power outages, impossibility of classes
			A_42_	Placement of classrooms near strategic objects
			A_43_	Lack of internet connection, impossibility of online communication
			A_43_	A state of frustration due to a constant flow of threatening information

**Figure 6:**
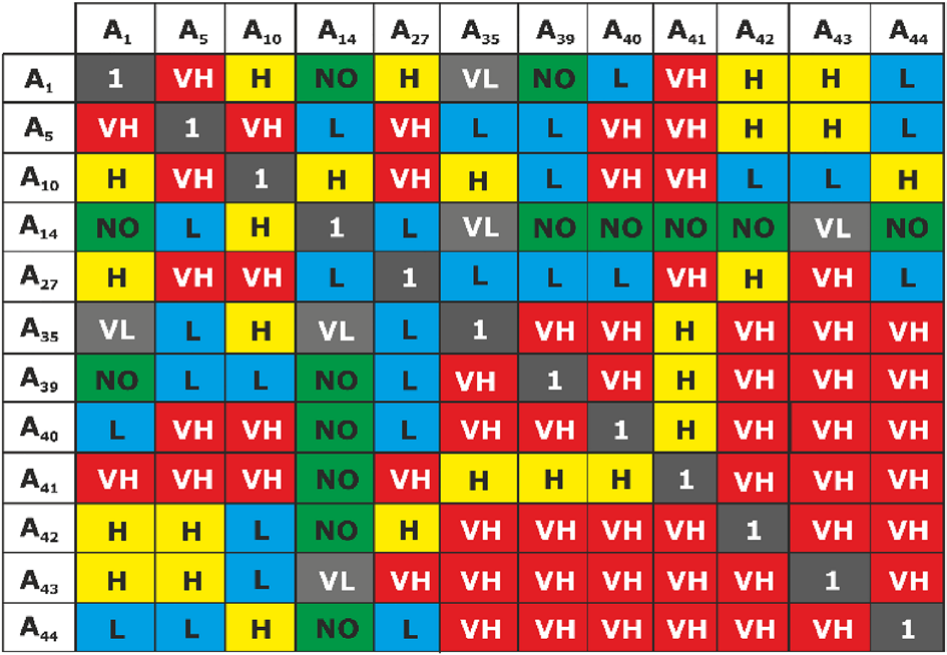
An example of a paired comparison matrix filled in by one of the experts.

Next, they are ranked using the DEMATEL method, where experts make paired comparison of the identified HFs, filling in the corresponding table (Fig. 6) in the Excel program. The dimensions of the matrix are determined by the number of HF (A12), which affect the appearance of a specific hazardous situation. Experts compile a matrix based on a subjective assessment of the greatest interaction with other indicators. In this case, the judgment is usually based on the interdependence of HF and inconsistencies. It is estimated that all HFs from each group will lead to a hazardous event and, in general, will harm the educational process. In addition, when compiling the matrix, experts are invited to take into account the possibility of controlling the manifestation of HF.

Further, the obtained results are processed using the appropriate mathematical apparatus, which is also performed in the Excel program, which allows obtaining HF prioritization (Table 14).

**Table 14:** Prioritization of HF based on degree of importance (r + c) and level of impact (r − c).

HF	Calculation data		Degree of importance of HF	Level of HF impact
	D	R	D + R	D − R
A_1_	28.44	28.73	58.36	0.8
A_5_	28.41	28.28	56.69	− 2.03
A_10_	28.41	31.21	57.17	− 1.79
A_14_	28.29	30.64	56.41	− 2.16
A_27_	28.02	28.39	58.93	1.15
A_35_	28.02	29.79	57.11	− 1.47
A_39_	27.94	30.42	57.85	− 1.06
A_40_	27.83	32.47	61.69	2.89
A_41_	27.65	30.21	60.29	2.04
A_42_	27.62	28.63	56.25	− 2.5
A_43_	27.83	33.37	61.2	2.54

As a result, on the basis of a detailed analysis of the impact of HF, we obtain their prioritization, based on establishing the degree of importance and the level of impact on the occurrence of a specific hazardous event. Thus, in the given example, considering the inconsistency of H_14_ “Teachers do not have appropriate qualifications”, it was established that the greatest influence is exerted by HF under the number A_40_, A_43_, A_41_, A_44_, A_27_, A_1_. At the same time, a negative sign in the impact level column indicates that they will have a greater impact on the magnitude of the consequences of the occurrence of a hazardous event. In a similar way, the FF analysis is conducted.

**Figure 7:**
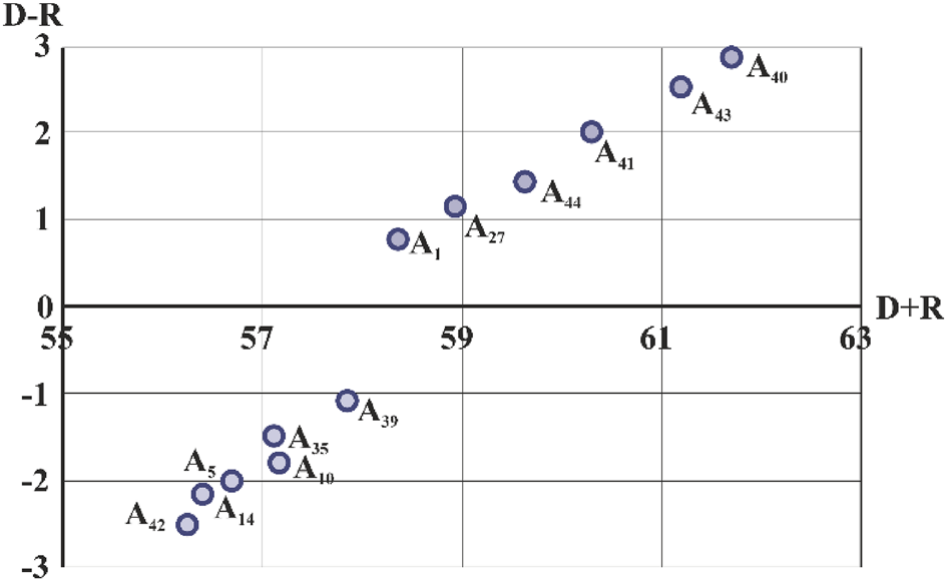
An example of a map of the connection between HF measurements.

The constructed prioritization of HF compatible with their ranking allows obtaining a map of connections between HF dimensions (Fig. 7) that visually shows their interdependence.

Determining the causal HFs and, as a consequence, FFs obtained during a similar procedure using the DAMTEL method allows moving to the step of calculating the risk from exposure to significant (causal) HFs (Table 15) and FFs (Table 16). As a result, we obtain risk values which, under certain conditions, that is, when hazardous and favorable factors coincide, can compensate each other, thereby reducing the risk level in quality management systems. The issue of interaction between various factors is not simple, as it requires careful verification and the experience of experts who will conduct the appropriate analysis. Of course, specific recommendations for compensating for negative factors with favorable ones can be made only in case of a thorough study of organizational culture, understanding of social-psychological phenomena occurring in the company: relationships, connections, beliefs, politics, activities and relationships of groups^43^.

**Table 15:** An example of a form for the initial assessment of occupational risks from HF.

Identification			Identification of HF, hazardous actions and inactions		The primary analysis is determination of the OR level for each HF and the overall OR hazard	
Hazard	Hazardous event	Negative consequences	HF	Probability of occurrence of a hazardous event from HF-*i*	The severity of the occurrence	Probability of occurrence of a hazardous event from HF-*i*
H_14_. Teachers do not have appropriate qualifications	Loss of accredit-tation	Loss of income from training	A_1_. Inadequate psychological state of teachers and students	4	5	20
			A_27_. Ineffective institutional support for employee welfare	3	5	15
			A_40_. Missile threats that cause neurotic states	3	4	12
			A_41_. Power outages, impossibility of classes	5	5	25
			A_44_ A state of frustration due to a constant flow of threatening information	4	5	20
			A_43_. Lack of internet connection, impossibility of online communication	5	5	25
			The overall primary negative OR of hazard j from all n HFs			122

**Table 16:** An example of assessing the risks from FF.

Identification			FF identification	The primary analysis is determination of the OR level for each FF and the overall OR hazard		
Probability	Favorable event	Positive conse-quences	FF	Probability of a favorable event occurring from FF-*i*	Degree of positive consequences from a favorable event occurring from FF-*i*	Level of *P*^+^_*i*_ from FF-*i*
H14. Teachers do not have appropriate qualifications	Accredi-tation	Revenue share loss	B_37_ Efficient mechanisms for improving educational programs	4	3	12
			B _38_ Perfection of the educational process planning system	5	3	15
			Total risk *j* from all *n* of FFs			*P*^+^ = ∑*P*^+^ = 27

Since the total negative risk *j* from all *n* of HFs is equal to 122 (see Table 15), and the total positive risk *j* from all *n* of FFs is equal to 27 (see Table 16), in this case, taking, as a rough approximation, a linear relationship between the impact of HFs and FFs, the total risk in the quality management system will be equal to:

$$ P = P^{ - } - P^{ + } = \sum {P_{i}^{ - } } - \sum {P_{i}^{ + } } = 122 - 27 = 95. $$

Although the proposed approach is not perfect, it does provide an overall measure of risk based on an integrated process that reflects the constructive confrontation of opposing factors. As a result, a creative solution to reduce the risk level is generated in the form of, perhaps even a new idea, as it becomes possible to comprehensively analyze the cause-and-effect relationships between the inconsistency and specific causal hazardous and favorable factors. Moreover, the proposed approach makes it possible to determine the most influential factor and further work with it: in case of a negative impact—by determining precautionary or preventative actions, and in case of a favorable factor—ways to enhance its influence.

The basic requirement for applying this approach is the need to adhere to standard academic training protocols, such as compliance with all rules and assumptions made, a clear basis for all choices, etc. In addition, the educational environment should meet reliability and validity criteria. The reliability requirement here refers to the degree to which a risk assessment produces the same results when analyzed repeatedly, and the validity requirement refers to the degree to which a risk assessment describes the specific concepts that it attempts to describe. Having accepted these criteria, results (beliefs), the value of the calculated risk level can be considered to a certain extent as justified^44^.

## Discussion

In the described process of risk management, the most important component is the identification of inconsistencies (hazards) and HF, which involves a thorough investigation of the cause and sources of risk, as well as events and situations that can significantly affect the overall results in relation to the goals of the educational process.

It is expected that risks will be studied and predicted at each stage of the educational process, based on key performance indicators established in the higher education quality assurance system, which is also emphasized in the research paper^27,45^. Such an approach requires systematic effort on identifying inconsistencies by constructing a monitoring system that assesses risks at specified intervals and facilitates the revision of precautionary measures depending on the current situation. A similar approach is implemented in Ref.^32^, but the difference of the proposed one is the involvement of a wide range of interested parties, which is one of the most important conditions for the formation of a high-quality educational process^46–49^. It is through the elaboration of both external and internal inconsistencies, and most importantly, proposals (opportunities), there are real ways to substantiate relevant managerial decisions based on the financial capabilities of educational institutions^27,50^.

Taken together, identifying inconsistencies of hazardous factors, opportunities, creating a register of them that will be constantly updated, identifying significant threats (for example, due to a pandemic, military operations, economic challenges, natural disasters)^51,52^, constructing cause-and-effect relationships between a threat (including in relation to the educational environment, teaching methods, etc.)—a hazardous event (for example, loss of accreditation, low-quality educational services, etc.) and consequences, taking into account all additional components (significant hazardous factors, opportunities), is the foundation for conducting the justification of effective precautionary and protective measures^53,54^, and most importantly, readiness for changes and upcoming challenges^54–56^. It is the construction of such a system that will make it possible, in the future, based on the construction of even the simplest models^57,58^, to predict the development of events and ensure readiness to respond to challenges^59,60^. The more detailed the current state that has developed at a specific point in time in the quality system of a conditional university^61,62^ is analyzed, the more opportunities there are for adopting appropriate ways of developing the educational environment, meeting the needs of interested parties, and most importantly, creating a safe environment, the potential of which will reveal the opportunities of participants in the educational process (both teachers in terms of ensuring academic freedom, and applicants, if possible, to reveal the relevant abilities that will be in demand in the labor market).

Despite the significant proposed approach publicity, it has significant advantages over the existing ones, as it allows a more thorough study of the occurrence of a hazardous event from the combined action of several factors. In addition, when compiling appropriate registers, experts analyze all the causes of the hazardous event occurring, assess the effectiveness of existing monitoring tools, which is the basis for savings through the redistribution of already existing resources^13^.

The strengths of the above approach include an integrated approach to the implementation of corrective actions, which is expressed in comprehensive monitoring and assessment of results, partnership among all interested parties, taking into account the impact of negative and favorable factors on the risk level. In addition, this allows analyzing the processes at the university in terms of the effectiveness of their functioning, which helps to create conditions for systematic educational process management, as opposed to intuitive control (based on tradition, trial and error).

Given that the risk management process is defined as “a planned and structured process aimed at improving the effectiveness of decision-making to ensure the appropriate activity of higher education institutions at a certain time^63^, there is a need to understand their consequences, which can be done through the construction of cause-and-effect relationships between challenges and possible hazardous events. In this case, to determine the “probability” of the hazardous event occurring, it is important to use a multivariate analysis^33,64^. Currently, there are powerful quantitative methods for assessing risks, such as Bayesian networks, but they are much more complex than the Bowtie method, and on the other hand, they require appropriate additional research to identify the relationships between various hazardous factors. We believe that Bayesian models are the next step in improving quality systems, since artificial intelligence can be used to perform the appropriate analysis.

Despite the considerable proposed approach is quite cumbersome, it has significant advantages over the existing ones, as it allows for a more thorough investigation of the occurrence of a hazardous event^65^ from the combined action of several HFs. In addition, during the compilation of relevant registers, experts analyse all the reasons for the occurrence of a hazardous event, evaluate the effectiveness of existing control measures, which is the basis of savings due to the redistribution of already existing resources.

It should be noted that most of the relevant HFs are interdependent, which is also taken into account during the implementation of the given approach. The more relationships are established, the higher the score will be compared to other HFs.

In general, referring to the requirements of the international standard ISO 9001:2015, there is a need to introduce a policy of the quality management system of higher education under martial law, which is based on the culture and traditions of the higher education institution, a set of beliefs and values that determine the behavior of the educational institution, which is aimed at minimizing risks^66^.

The significance of the presented research lies in its practical use in educational institutions facing the choice of the optimal methodology for applying a risk-oriented approach in education quality management systems. The shortcomings of the study include the influence of subjectivity at the first stages of the risk management process, which is characteristic of expert methods, when forming from the relevant registers, determining the dependence between inconsistency and HF. However, in the future, the application of a mathematical approach to risk ranking will allow us to identify inappropriate relationships and return to the revised previous steps of HF identification, which are associated with a specific inconsistency.

## Conclusions

The improvement of the risk management process in the system of higher education of students under martial law is based on the sequence of six main steps and differs from known processes by the presence of the identification of the cause-and-effect relationship “hazard (inconsistency)-hazardous event-consequences”, identification of HFs and FFs (hazards-inconsistencies) of the internal and external environment, which affect the probability and/or degree of severity of a hazardous event—the occurrences of inconsistency, which is carried out after determining the inconsistency; determination of causal (ignoring consequential) HFs and FFs by an acceptable method (for example, DEMATEL or ANP).

The developed and described registers of inconsistencies (hazards), as well as HFs and FFs, based on the requirements for accreditation of educational programs and the international standard ISO 9001:2015, etc., using a risk-based approach, provide the basis for transforming the goals of a higher education institution in conditions of martial law in order to guarantee the effective implementation of the mission and strategy of the relevant requirements.

It is proposed to determine the criteria for risk analysis, which involves establishing the probability and degree of severity of a hazardous event from the action of the causal HFs and FFs impact, to use the cumulative effect of the causal HFs, as well as FFs, which is calculated as the sum of their effects.

Flexible forms that can be used by institutions of higher education are offered for the initial assessment of negative risks of HF hazards, prioritization of HFs and FFs based on the degree of importance and level of impact, analysis of inconsistencies as a result of the examination of a conditional educational program, a questionnaire to establish the impact of HFs and FFs on a specific inconsistency without changes or after adaptation in accordance with the specifics of the relevant higher education institution.

## Data Availability

All data generated or analyzed during this study are included in this published article.

## Abbreviations

DE-MATEL: Decision making trial and evaluation method
QM: Quality management
HF: Hazardous factors
AHP: Analytic hierarchy process
ANP: Analytic network process
GF: Group of factors
HF: Hazardous factors
HE: Hazardous event
VH: Very high impact
H: High impact
L: Low impact
VL: Very low impact
NO: Zero impact
OR: Occupational risks
FF: Favorable factor
HF: Hazardous factor
